# Editorial: Recent advances in organ-sparing techniques in pediatric solid tumor surgery

**DOI:** 10.3389/fped.2023.1247287

**Published:** 2023-07-07

**Authors:** Chan Hon Chui

**Affiliations:** Surgery Center for Children, Mount Elizabeth Hospital, Singapore, Singapore

**Keywords:** pediatric tumor surgery, organ-sparing techniques, good oncological outcome, wilms tumor, hepatoblastoma

**Editorial on the Research Topic** Recent advances in organ-sparing techniques in pediatric solid tumor surgery

In recent years, the practice of pediatric solid tumor surgery has evolved with significant emphasis on organ-sparing techniques. While the surgical goal for good oncological outcomes remains paramount, the ability to retain the patient's innate organ function has been realized as new surgical techniques emerged. A fine collection of manuscripts on nephron-sparing surgery (NSS), the application of indocyanine green (ICG) fluorescence imaging navigation for surgery in pediatric renal tumors, ex-vivo tumor dissection followed by kidney autotransplantation in bilateral Wilms tumor (WT), surgical treatment of postoperative intractable bile leakage after liver tumor surgery, hepatectomy with Rex bypass for a child with hepatoblastoma and portal vein thrombosis, and liver resection for hepatoblastoma using ICG fluorescence and water-jet dissector (WJD) have been presented. Such treatment techniques improve our children's long-term survival and quality of life.

NSS for WTs has been reviewed and discussed by Murphy et al. The technical aspects of the conduct of *nephron-sparing surgery for synchronous bilateral WT*, including the recent advances in the use of imaging adjuncts such as pre-operative three-dimensional (3D) imaging and intraoperative fluorescence-guided surgery were discussed. Such detailed preoperative and intraoperative anatomic planning and mapping of bilateral WT has been shown to increase the chance of successfully performing bilateral NSS. Feng et al. reported on their initial experience with the *clinical application of ICG fluorescence imaging navigation for pediatric renal tumors*. In a feasibility study of ICG in renal tumors in children, the authors hypothesized that renal tumors have differential ICG uptake from the normal kidney, an experience similar to adult renal cancer. Significantly, different fluorescence between the tumor and normal kidney allowed the operator to identify the tumor and its boundary, and to complete NSS while ensuring the negative margin. This fluorescence localization technique also makes it possible to visualize the sentinel lymph nodes, providing the possibility for accurate sentinel lymph node resection and adequate lymph node sampling in the future. Zhong et al. described their experience in *ex-vivo tumor dissection followed by kidney autotransplantation in bilateral WT*. Challenging tumors for a traditional NSS including large tumors, mid-pole tumors, tumors close to the renal hilum, those unable to preserve sufficient normal residual renal tissue, and those where major intra-operative bleeding risk may affect the judgment of the resection margin, may benefit from this technique. Intra-operative tumor rupture and dissemination may be avoided when tumor resection is performed in an isolated bloodless state Thus, this can be considered an alternative approach for cases of similar complexity.

*Surgical treatment of postoperative intractable bile leakage after liver tumor surgery in children* was reported by Han et al. Bile leakage, one of the most common complications after liver tumor surgery, leads to postoperative morbidities such as abdominal abscesses, longer hospital stays, and higher postoperative mortality due to liver failure. When conservative treatment fails, bilio-enteric anastomosis and bilio-cholecyst anastomosis may be used as described in this report. *A case report on hepatectomy with Rex bypass for a child with hepatoblastoma and portal vein thrombosis* was presented by Kitagawa et al. Pediatric liver tumors with portal vein obstruction are often candidates for liver transplantation. However, lifelong use of immunosuppressants and invasiveness to healthy donors in the case of living donor liver transplantation is inevitable. This report illustrated that in the right lobe of the liver tumor, complete resection of the portal vein trunk may be possible by creating a Rex bypass, with end-to-side anastomosis to the umbilical portal vein. *Minimal tissue damage and low coagulation liver resection for hepatoblastoma using ICG fluorescence and water-jet dissector* (WJD) were reported by Onishi et al. NIR fluorescence imaging with ICG was applied to visualize the line of liver resection and WJD was for liver parenchymal dissection in pediatric hepatoblastoma. The combined use of NIR imaging and WJD was deemed to be useful for pediatric hepatectomy.

## Publisher's note

All claims expressed in this article are solely those of the authors and do not necessarily represent those of their affiliated organizations, or those of the publisher, the editors and the reviewers. Any product that may be evaluated in this article, or claim that may be made by its manufacturer, is not guaranteed or endorsed by the publisher.

